# Alcohol Consumption by Italian and Spanish University Students in Relation to Adherence to the Mediterranean Diet and to the Food Neophobia: A Pilot Study

**DOI:** 10.3390/healthcare10020393

**Published:** 2022-02-18

**Authors:** Paola Aiello, Ilaria Peluso, Débora Villaño Valencia

**Affiliations:** 1 Health Sciences PhD Program, Universidad Católica de Murcia UCAM, Campus de los Jerónimos n°135, Guadalupe, 30107 Murcia, Spain; paola.aiello@uniroma1.it; 2 Department of Physiology and Pharmacology “V. Erspamer”, La Sapienza University of Rome, 00185 Rome, Italy; 3 Council for Agricultural Research and Economics, Research Centre for Food and Nutrition, Via Ardeatina 546, 00178 Rome, Italy; 4 “Nutrición, Estrés Oxidativo y Biodisponibilidad” Research Group, Faculty of Health Sciences, School of Pharmacy, Universidad Católica de Murcia UCAM, Campus de los Jerónimos n°135, Guadalupe, 30107 Murcia, Spain; dvillano@ucam.edu

**Keywords:** alcohol, Mediterranean diet, university students, food neophobia, dietary habits, lifestyle

## Abstract

This work aimed to relate alcohol consumption with adherence to the Mediterranean diet (MD) and with food neophobia (FN) among Italian and Spanish university students. Volunteers (*n* = 194, 108 Italian and 86 Spanish), recruited at the La Sapienza University of Rome and the Catholic University of Murcia, filled in standardized questionnaires to evaluate alcohol consumption (AUDIT), FN (FN Scale: FNS), and adherence to the MD (MDS-14, MED-55, QueMD). In addition to the previously reported QueMD sub-score (aMED), a sub-score for non-typical MD foods (ntMED, carbonated and/or sugar-sweetened beverages (soft drinks), butter, margarine, or cooking cream, and manufactured sweets, pastries, and cakes) was evaluated. Italian females had higher MED-55 and FNS scores, and a lower AUDIT score than Spaniards (*p* < 0.01). Students who stayed with their family (resident) were more adherent to MD than those who moved away from home. Resident Italians consumed less beer, hard liquors, and cocktails than Spaniards on Saturday nights (*p* < 0.01). There were negative correlations between AUDIT and QueMD (R squared: 0.137, *p* < 0.05), and AUDIT and ntMED (R squared: 0.201, *p* < 0.01) in Spaniards, however, there was no relationship between AUDIT and other MD scores. In conclusion, this pilot study suggests that non-typical MD foods and Saturday night consumptions, related to being far from home, have a great impact on alcohol consumption.

## 1. Introduction

In recent years, the consumption of alcohol among university students has become a problem of health concern at an international level [1,2]. As reported in the Global Status Report on Alcohol and Health prepared by the World Health Organization (WHO), on average about 2.3 billion people in the world consume alcoholic beverages [3]. A report by the Italian Ministry of Health from 2015 highlighted that 64.5% of Italians over the age of 11 years consumed an alcoholic beverage at least once in their life, with a clear majority among males compared to females [4]. Furthermore, it was reported that between 11 and 24 years the consumption of alcoholic beverages occurred often outside meals [4]. According to the OECD (Organization for Economic Co-operation and Development), Spanish women with high education are more likely to be hazardous drinkers than less educated women, while men with low education are more likely to drink at risk [5]. College students report a greater increase in drinking than their peers who do not attend colleges and universities [6]. In addition to alcohol abuse, binge drinking, especially on weekends, is an increasing phenomenon among university students [7]. At the European level, it has emerged that two-thirds of university students are high-risk drinkers [8]. There are marked differences in the type of drink consumed. Worldwide, 44.8% of alcohol is consumed in the form of spirits, the most consumed beverage in the regions of Southeast Asia and the Western Pacific, followed by beer (34.3%) and wine (11.7%) [4]. In the last 20 years, the portion of liters of wine consumed has decreased, while beer has increased, in Italy [4]. In 2016, 54% of alcohol per capita consumption among the Spanish population aged 15 years and older was of beer, followed by 28% of spirits, and 18% of wine [9]. Although the daily consumption of moderate quantities of red wine is a well-established custom of the eating habits of the Mediterranean area [3], the adverse effects remain predominant [10]. Moreover, it has been recently pointed out that messages on the benefits of moderate wine drinking should be avoided, also considering the other sources of polyphenols in the Mediterranean diet (MD) [11]. Besides, the perceived utility of warnings on alcoholic beverages in Italy was high among university students with moderate intake, but low among those with an at-risk consumption [12]. When the compliance with the recommendations of the MD pyramid was evaluated among Italians, the regular consumption of fruits and vegetables was significantly more common in females, and it increased with age and education (84.7% of those with a university degree) [13]. Controversial results came from studies that evaluated the relationship between alcohol consumption and adherence to the MD in Spain [14,15,16]; however, in addition to high alcohol intake, factors that predicted a worse diet in Spanish university students included male gender and living alone [17]. The university population is divided into two categories: those who live with their parents and those who live away from home [18]. The reasons that influence food choices made by students include a change in lifestyle, the convenience of fast-food, and the taste [18]. It has been recently reported that familiarity with coffee/tea characterized by a higher bitterness and astringency was lower in individuals high in food neophobia (FN) [19]. The latter has been defined by psychologists as the reluctance to eat unfamiliar foods [20], and it was associated with both low adherence to MD [21] and low familiarity with vegetables, irrespective of their sensory properties, in Italian adults [19]. Nonetheless, FN correlated with a low vegetable consumption in Spanish schoolchildren [22]. This work aimed to relate alcohol consumption among university students to the FN and to the adherence to MD. The latter was assessed with validated questionnaires that assigned different scores to wine consumption.

## 2. Materials and Methods

### 2.1. Study Design, Recruitment, and Data Collection

This study is part of the multi-center cross-sectional observational study “Lifestyle, Self-medication and Use of Nutraceuticals in a Population of Italian and Spanish Students”, acronym STANIS, registered on ClinicalTrials.gov (Identifier NCT04099420). The study was carried out from July 2019 to September 2021. Undergraduate and doctoral students aged between 18 and 35 years were recruited through verbal disclosure, e-mails, and notice boards at La Sapienza University of Rome and UCAM. All the volunteers included in the study signed the informed consent, accompanied by an informative note, and the recruiter assigned them an alphanumeric code to guarantee privacy during the data processing and analysis phases. To collect information about eating habits and lifestyle, on-line standardized questionnaires were administered: Alcohol use disorders identification test (AUDIT) [23,24,25], Mediterranean diet score (MDS-14) [26], Mediterranean score (MED-55) [27,28], questionnaire to measure Mediterranean diet (QueMD) [29], and the food neophobia scale (FNS) [20]. A questionnaire was used to evaluate the consumption of several beverages (alcohol, coffee, and tea) and another questionnaire was administered to collect general information (age, sex, course degree, and residence: with family or away from home). Height and weight were measured with a SECA 217 portable stadiometer and balance OMRON BF511, respectively. The OMRON BF511 used the height information to calculate the body mass index (BMI) classification according to the values for obesity judgment proposed by WHO: underweight, BMI < 18.5 kg/m^2^; normal weight, BMI between 18.5 kg/m^2^ and 24.9 kg/m^2^; overweight, BMI from 25 kg/m^2^ to 29.9 kg/m^2^; obese, BMI ≥ 30 kg/m^2^.

### 2.2. Validated Questionnaires Data Analysis

AUDIT was used to assess alcohol consumption, drinking behaviors, and alcohol-related problems. It is a 10-item screening tool developed by the WHO. Items are selected to measure the three conceptually distinct dimensions of intake (items 1–3), dependence (items 4–6), and adverse consequences of drinking (items 7–10) [23]. Apart from the last two items, AUDIT questions allude to the previous year, and the score assigned to each item—that ranges from 0 to 4—is generally based on the frequency of occurrence. A score of 8 or more is considered to indicate hazardous or harmful alcohol use [24,25]. Nevertheless, a cut-off point of 6 has been suggested for women since the original score yielded lower sensitivities and higher specificities for women than for men. The differentiated cut-off scores would correct the gender discrepancies in the sensitivity of the measure [24]. Scores ≥ 16 are associated with harmful drinking for men and women, while scores ≥ 20 with alcohol dependence [23].

FNS was used to evaluate the reluctance to eat unfamiliar foods. FNS is a 10-item questionnaire with answers given on a 5-point agreement scale (1 = not at all descriptive of me and 5 = very descriptive of me), and the score can range from 10 to 50. Scores ≥ 28 were associated with food neophobia, between 12 and 27 were neutral, and less than 12 were associated with food neophilia [20].

To evaluate the grade of adherence to MD, three different questionnaires were administered to the volunteers.

MDS-14, validated in the PREDIMED study [26], is a 14-item questionnaire, of which 12 inquire on the frequency of consumption of typical components of MD (items 2–12, 14), while the other two (items 1, 13) concern dietary habits. Each item can be assigned a score of 0 (no adherence to MD) or 1 (adherence), with a total score ranging from 0 to 14 (≤5 is low adherence, between 6 and 9 is medium adherence, and ≥10 is high adherence).

MED-55 is another index proposed by Panagiotakos and colleagues [27,28] to estimate the adherence level to MD. It is an 11-item questionnaire that examines the frequency of consumption of the main components of MD. For the intake of items supposed to be close to this dietary pattern, scores 0, 1, 2, 3, 4, and 5 were assigned when volunteers reported no consumption, rare, frequent, very frequent, weekly, and daily, respectively; whereas, for the consumption of food assumed to be away from this pattern, the same scores were assigned on a reverse scale. The final score ranged from 0 to 55, with higher values indicating greater adherence to MD. A score of 55 represents 100% adherence to the Mediterranean dietary pattern and a score equal to k represents (k/55) × 100% agreement to this nutritional model. Five classes of distribution of the MED-55 score have been developed (0–11, 12–22, 23–34, 35–44, and 45–55) [27].

QueMD is a self-administered 15-item questionnaire [29], with a score ranging from 0 to 30, that includes questions for the nine food items considered as key components of MD (wholegrain cereals, raw or cooked vegetables, legumes, fresh fruits, dried fruits, red or processed meat, fish, wine, and olive oil), using two different questions to evaluate the intake of wholegrain cereals (wholegrain pasta or rice and wholegrain bread and substitutes). The alternate Mediterranean diet (aMED) score, ranging from 0 (minimal adherence to MD) to 9 (maximal adherence), was calculated from QueMD assigning 1 point to volunteers reporting consumptions above the average levels for each of the nine foods that are characteristic of MD [30]: vegetables and fresh fruits (≥2/day), dried fruits (≥2/week), wholegrain cereals (≥1/day), pulses and fish (≥2/week), and olive oil (≥3/day) intakes. Moreover, 1 point was also assigned to those consuming red and processed meat ≤ 1–3/week, and for men drinking 1–2 glasses of wine per day (corresponding to 125–250 mL) or women drinking a limited amount of wine (1/2–1 glass/day, corresponding to 62.5–125 mL) [30]. Additional items are the consumption of white meat (chicken, turkey, and rabbit) and dairy products, focusing only on milk and yogurt (excluding cheese). Furthermore, questions for three additional food groups that are not typical of MD are included: carbonated and/or sugar-sweetened beverages (soft drinks), butter, margarine, or cooking cream, manufactured sweets, pastries, and cakes. In the present study, a sub-score for these non-typical MD foods was evaluated (ntMED). For each of the food items, participants could choose between five consumption levels, ranging from “never or seldom” to a high frequency. For each question, a standard portion was indicated to help report consumption as correctly as possible [29].

### 2.3. Statistical Analysis

A descriptive statistical analysis (averages, standard deviations, percentages) was first performed. The normal distribution of variables has been checked by the Shapiro–Wilk test. Categorical variables have been expressed as percentages, while continuous variables were expressed as means with standard deviation (SD) or medians with interquartile intervals. Linear regression was used to evaluate relationships between variables. Bivariate analysis was conducted using an unpaired t-test and Mann–Whitney test, for continuous variables, and chi-square test and Fisher’s exact test, for categorical variables. Statistical analysis was performed using the Graph Pad software (GraphPad Prism 8 XML ProjecT, La Jolla, CA, USA). Variables with a *p*-value lower than 0.05 were considered statistically significant.

## 3. Results

### 3.1. Participants

Participants, predominantly females (Table 1), were university, doctoral and postdoctoral, specialization and master students from both scientific (81.2%) and humanistic (18.8%) areas.

Regarding BMI, it was significantly higher in Spaniards (*p* < 0.05) among normal-weight students (Table 1). However, no significant differences have emerged in the percentages of underweight (7.4% of Italians and 8.2% of Spaniards) and obese (7 Italians and only one Spanish subject), whereas a similar prevalence of overweight was observed (15.7% of Italians and 16.3% of Spaniards).

### 3.2. Consumption of Alcohol and Caffeinated Beverages

Frequencies of the consumption of coffee, tea, and alcohol showed no significant differences, both between Italian and Spanish subjects and between males and females. Among Italian students, 89.6% consumed coffee, 59.4% tea, and 86.8% alcoholic beverages. For Spanish students, 71.4% stated to consume coffee, 52.4% tea, and 74.6% alcoholic drinks. Among subjects who reported alcohol consumption, the type and quantity consumed on a weekday and Saturday night did not differ significantly (Table 2).

On the other hand, when we stratified alcohol consumption, classifying it according to the place of residence of the university students, differences between Italian and Spanish resident students in the number of hard liquors and cocktails consumed, irrespective of the weekday or Saturday night pattern, were statistically significant (Table 3), as well as beer consumption on Saturday night.

Despite the overall wine consumption not being significant among groups, non-resident Italian female students reported consuming only wine on Saturday night in quantity comparable to the resident female students (1.2 ± 0.4 and 1.5 ± 0.8, respectively); whereas, among Spanish women, the consumption of wine on weekends is higher in students who have moved away from home compared to the resident ones (wine: 1.8 ± 1.1 vs. 1.2 ± 0.5). During a weekday, both female and male students consumed only wine and beer, in Italy, regardless of their residence (around 1 portion), whereas Spanish females who moved away from home tended to consume more wine than resident ones (1.4 ± 0.9 vs. 1.2 ± 0.4), and male students consumed less wine (around 1 portion), regardless of the residence.

### 3.3. Dietary Habits and Adherence to Mediterranean Diet (MD)

**Figure 1:**
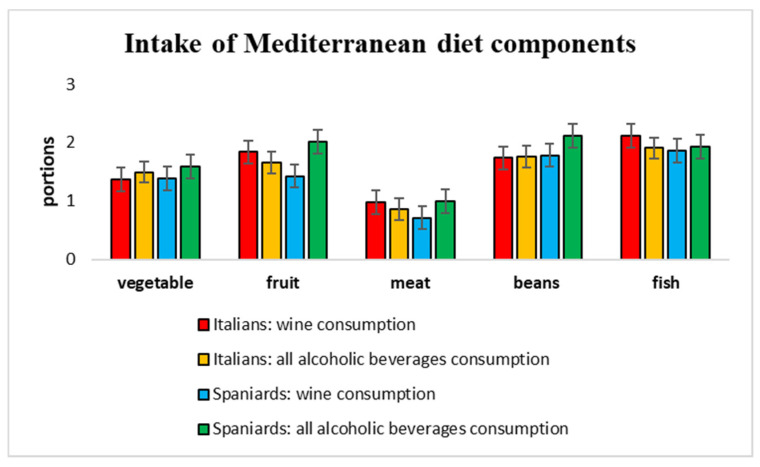
Consumption of the main components of the Mediterranean diet among drinkers. For each category of a food item, the average number of portions consumed by Italians and Spanish drinkers of only wine, or all alcoholic beverages, are reported. Data are means with standard deviation.

Regarding eating habits, most of the population studied (79.4%) stated to not follow any particular diet (86.8% of Italians and 70.2% of Spaniards), 4.6% stated to follow a lactose-free diet (5.7% of Italians and 1.9% of Spaniards), and 2.9% a gluten-free diet (2.8% of Italians and 1.9% of Spaniards). Among Italian students, there were subjects who followed a diet low in lipids and carbohydrates (0.9%), and a maintenance diet of 1700 kcal (0.9%); whereas, only among Spanish students were participants who went on a vegetarian (2.8%), vegan (2.8%), and protein (0.9%) diet. Figure 1 reports the average consumption of fruit, vegetable, red meat, fish, and beans, grouped according to the consumption of only wine or all alcoholic beverages. Differences between the two groups for Italian and Spanish subjects were not statistically significant.

Most of the students did not follow the MD pyramid recommendations for the consumption of vegetables (≥2/day), fruit (≥3/day), red meat (<1/day), beans (≥3/week), and fish (≥3/week). Differences between males and females, and Italian and Spanish subjects were not statistically significant; however, when data were stratified by place of residence, significant differences between Italians and Spaniards were observed among resident students (Table 4).

Although less than half of the Italian students who stayed with their family consumed the recommended portions of MD, they were more adherent to MD than those who moved away from home. We hypothesized that FN could be higher in students with higher MD adherence. Although no significant relationship was found among FNS and the different MD scores, FNS was higher in Italians compared to Spaniards (Table 5). From the FNS questionnaire, it emerged that 5.8% of the Italian sample had a maximum predisposition to taste new foods, and 26.2% (23.8% of males and 27.9% of females) had a greater reluctance to try new foods. Regarding Spanish students, 6.5% (4.8% of males and 7.3% of females) had food neophilia, and 14.5% (9.5% of males and 17.1% of females) have reported a score higher than the cut-off point of 28. Differences between FNS of Italian and Spanish females were statistically significant (Table 5).

**Figure 2:**
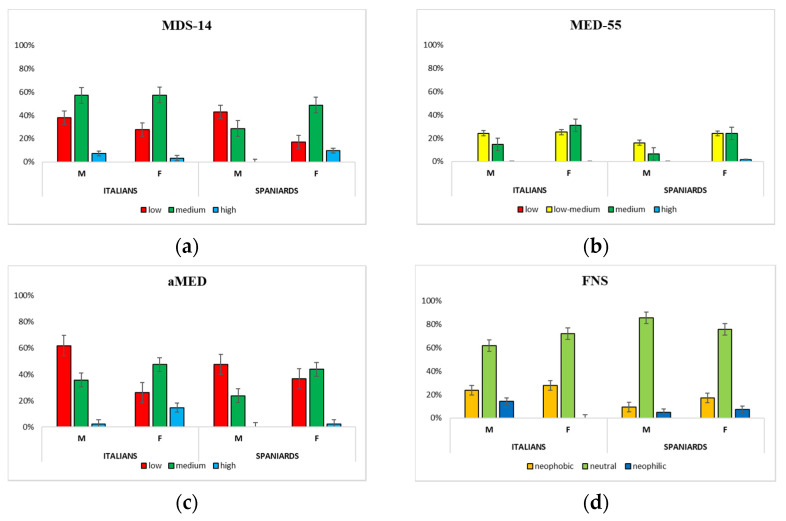
Distribution of the sample based on the adherence level to Mediterranean diet (MD) and the presence of food neophobia (FN). FNS: Food neophobia scale; MDS-14: Mediterranean diet score; MED-55: Mediterranean score; aMED: alternate Mediterranean diet score. Percentages of Italians and Spaniards who presented a low, medium, and high adherence grade to MD according to the MDS-14 (**a**), MED-55 (**b**), and aMED (**c**) scores; (**d**) Percentages of Italians and Spaniards who were neophobic, neutral and neophilic, according to the FNS.

Medians of AUDIT scores (Table 5) were below those indicating hazardous or harmful alcohol use (cut-off points of 8 and 6 for men and women, respectively). However, 15.5% of Italians and 30.7% of Spaniards showed hazardous alcohol use (14.3% of males and 25.5% of females) (*p* < 0.001). In Figure 2, the percentages of students with low, medium, and high MD adherence, assessed with MDS-14, MED-55, and aMED, are shown.

Among Italian and Spanish subjects, only 4.9% and 6.5%, respectively, showed high MD adherence with MDS-14. Regarding MED-55 scores, none of the Italian and Spanish males showed a score between 45 and 55, which represented the maximum level of adherence to MD. However, the difference in MED-55 scores between Italian and Spanish students was statistically significant (Table 5).

Regarding QueMD, differences between Italian and Spanish students (both males and females) were not statistically significant. According to aMED scores, differences between Italian and Spanish subjects, and Italian males and females were statistically significant (Table 5).

**Figure 3:**
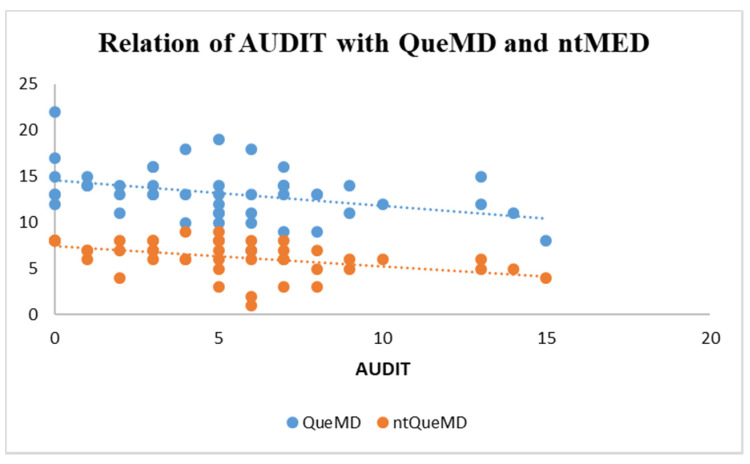
Linear regression between AUDIT score and adherence level to the Mediterranean diet according to QueMD questionnaire and the sub-score ntMED, in the Spanish sample. AUDIT: Alcohol use disorders identification test; QueMD: Questionnaire to measure Mediterranean diet; ntMED: non-typical MD foods score. AUDIT versus QueMD: blue trendline; equation: y = −0.2789x + 14.612; R squared: 0.137; 95% CI: −0.489 to −0.069; *p* < 0.05; AUDIT versus ntMED: orange trendline; equation: y = −0.2172x + 7.421; R squared: 0.201; 95% CI: −0.347 to −0.087; *p* < 0.01.

A significant difference in ntMED, between Italians and Spaniards, was not found. However, linear regressions, whose data are shown in the legend of Figure 3, reported negative correlations both between AUDIT and QueMD and between AUDIT and ntMED in Spanish students (Figure 3), but no relationship has emerged between AUDIT and the other MD questionnaires both in Italian and Spanish subjects.

## 4. Discussion

This study aimed to relate AUDIT with FN and adherence to MD, assessed with validated questionnaires that assigned different scores to wine consumption, in two countries of the Mediterranean basin, Italy and Spain, among university students. In both countries, the average AUDIT scores were below those indicating hazardous or harmful alcohol use (cut-off points of eight and six for men and women, respectively). However, 15.5% of Italians and 30.7% of Spaniards showed hazardous alcohol use. A recent study conducted in Spain reported that 26.2% of participants were categorized as high-risk drinkers, according to the AUDIT questionnaire [15]. Carlos et al. [14] found that 17.6% of the recruited Spanish university students may be considered at risk of developing alcohol dependency, and 2.4% can be considered as having alcohol dependence syndrome (ADS). López-Moreno et al. [15], according to the AUDIT score, categorized 26.2% of Spanish university students as high-risk drinkers, and 7.7% as suffering from ADS [15]. In our study, the safe consumption of alcohol can be due to the habits of our sample, since 77.4% of Italians and 73.0% of Spaniards stated to generally stay at home in the evening, while 22.6% of Italians and 27.0% of Spaniards went out. It was customary to have an aperitif before dinner (62.3% of Italians and 57.1% of Spaniards). Worldwide, most alcohol is consumed in the form of spirits, while in Italy the portion of liters of beer has increased and that of wine has decreased [4]. In our sample, the main alcoholic beverage used was beer, consumed by 60.9% of Italians and 76.6% of Spaniards.

Participants were asked to specify the type of alcoholic beverage consumed and the frequency of consumption in a day, to correlate the consumption of alcohol, especially red wine, with the adherence to MD. Previous studies have demonstrated that young adults did not follow national recommendations [31,32]. In line with what has been reported by OECD [33], females had better dietary habits than males did. We observed that Italian university students who live with their parents eat much more fruit, vegetables, fish, and legumes, whereas the departure from the ideal MD model seems much more pronounced among the non-resident students. This choice can be attributed not only to their greater independence but also to the economic limitations that push them to spend less on the purchase of food [18]. In fact, they tend to consume high-energy-density nutrient-free foods that are cheaper than fruit and vegetables [34,35]. In our study, Italian females had a higher FNS and lower AUDIT score, compared to Spanish ones.

Contrary to Scholz et al. [36], in the present study, university students who consumed only wine did not have greater adherence to MD than other volunteers. Although Minzer et al. [37] suggested that moderate wine consumption with meals is a positive item in the MD score, among the three differently used validated questionnaires, only aMED assigned a gender-specific moderate wine consumption [29,30]. MDS-14 assigns a greater score to a higher quantity of wine consumed [26], whereas MED-55 assigns a higher score to the lower consumption of alcoholic beverages, regardless of type [27,28].

In our study, only two Italians and none of the Spaniards stated to consume at least a glass of wine per day (the minimum required to comply with the MSD-14 score [26]). However, 86 Italians and 53 Spaniards have reported an intake of less than 300 mL of alcoholic beverages per day, which is the maximum required to be considered adherent to MD, according to the MED-55 score [27]. In the study conducted by López-Moreno et al. [15], the questionnaire from the PREDIMED study (MDS-14) was used, and it was reported that a high percentage of the students did not reach the intake of wine for the assignment of one point. On the other hand, Rodríguez-Muñoz et al. [16] and Carlos et al. [14] used the Mediterranean diet quality index for children and adolescents (KIDMED), not including alcoholic beverages, to evaluate the relationship between hazardous alcohol use and MD adherences in Spanish university students. Rodríguez-Muñoz et al. [16] reported that the students who declared eating fruit or drinking fruit juice every day, appreciating pulses, and using olive oil, had a lower tendency to drink heavily, assessed with AUDIT. Carlos et al. [14] found no relationship between the level of alcohol consumption and the degree of adherence to MD [14]. Accordingly, we found no relevant differences between the preference for alcoholic beverages and adherence to MD with MDS-14 and MED-55. The low relationship may be due to the consumption of other alcoholic beverages. In particular, 56.5% of Italians and 53.2% of Spaniards have reported drinking a bottle of beer (33 cl) within a day, 4.4% of Italians and 12.8% of Spaniards consumed two bottles within a day, whereas only one Spanish female student declared to consume eight bottles of beer within a day. Furthermore, 22.8% of Italians and 22.5% of Spaniards consumed hard liquor (40 mL), whereas 29.35% of Italians and 17.0% of Spaniards consumed an alcoholic cocktail (40 mL). There is an inconsistency between the MED-55 and the food pyramid proposed by Vitiello et al. [38] regarding the score assigned to the intake of red meat and dairy products. Furthermore, the MDS-14 includes the negative consumption of butter, which is not contemplated in the MED-55, that takes into consideration the consumption of milk and derivatives, assigning a lower score for a high frequency of consumption. However, due to the low score of MD adherence assigned to alcohol consumption, regardless of the type of beverage, MED-55 can be considered healthier according to the latest Italian national guidelines for nutrition [39] and to the “*Healthy Hydration Pyramid*” of the Spanish Society of Community Nutrition (SENC) [40].

In the present study, on average, Italians had moderate MD adherence assessed as MED-55 and aMED. From the aMED (including vegetables, fresh fruits, dried fruits, wholegrain cereals, pulses, fish, and olive oil) it emerged that 37.6% of the sample had poor adherence to MD, whereas the analysis of MED-55 has revealed that no subject had low adherence to this dietary pattern. Significant differences in ntMED (including carbonated and/or sugar-sweetened beverages (soft drinks), butter, margarine, or cooking cream, and manufactured sweets, pastries, and cakes), between Italians and Spaniards, were not found. However, there were negative correlations between AUDIT and QueMD (*p* < 0.05), and AUDIT and ntMED (*p* < 0.01)—the score that includes foods not typical of MD (usually consumed on Saturday night among Spaniards)—but no relationship between AUDIT and others MD scores, including aMED. Therefore, no typical MD foods had the greatest impact on our sample, especially among Spaniards. In agreement, in the study conducted by Rodríguez-Muñoz et al. [16] the Spanish university students who declared going to a fast-food restaurant more than once a week, and eating sweets and candy several times every day, had a higher tendency to be at risk of excessive alcohol consumption [16].

This pilot study has both strengths and limitations. The major strength of our study is that we used different standardized questionnaires for the evaluation of MD adherence. Another point of strength is that we found a relationship only between ntMED and AUDIT in Spaniards, whereas Italians presented a high FN, suggesting a higher attachment to traditions. The study points to differences in dietary patterns at the household level between the two countries, which calls for further studies to elucidate.

The study also has several limitations and our results should be interpreted carefully. Participants were predominantly women and students of the scientific area; however, similar limitations were reported in a larger study [15]. The major limitation of our study is the limited sample size. Therefore, the generalization to the whole population of university students cannot be accomplished. However, moderate adherence to MD was observed, and females had a higher adherence to MD (assessed with aMED) than males, in accordance with other studies [41,42,43,44]. Unfortunately, the COVID-19 pandemic has represented a limitation to this study since it has induced strong changes in the lifestyle and eating habits of university students and limited recruitment. Furthermore, due to the COVID-19 pandemic, students have reduced their quantity of drinking owing to the closing of the pubs, and returning to live with their parents may have been a protective factor for heavy drinking [45,46]. On the other hand, students who presented more symptoms of depression and anxiety during lockdown have reported an increased alcohol consumption [47]. Therefore, further research is needed to demonstrate the association between alcohol intake and specific dietary patterns.

## 5. Conclusions

Overall, our study has found a medium adherence to MD and a low risk of excessive alcohol consumption. However, being far from home and the Saturday night consumption of alcoholic beverages, different from wine, can be risk factors for alcohol use disorders, assessed with AUDIT. The latter correlated with non-typical MD foods consumption. Questionnaires for the evaluation of MD adherence considered and/or assigned scores to wine consumption in a different way, and only MED-55 assigned a low score to alcohol consumption, regardless of the type of beverage. This must be kept in mind when comparing data from different studies, and the use of more than one score could be useful. In conclusion, this pilot study suggests that no typical MD foods consumption, rather than typical MD intake, had the greatest impact in our sample, and that Saturday night consumption and being far from home can be risk factors for excessive alcohol consumption.

## Figures and Tables

**Table 1:** Sample characteristics.

Variable	Italians (*n* = 108)		Spaniards (*n* = 86)	
	Male	Female	Male	Female
Sex (%)	42.6	57.4	40.7	59.3
Age	26.1 ± 3.7	25.6 ± 3.4	25.3 ± 2.8	24.5 ± 3.7
BMI				
Underweight	-	17.7 ± 0.4	16.3 ± 0.0	17.6 ± 1.2
Normal weight	22.5 ± 1.5	20.9 ± 1.6 ^†††^	23.2 ± 1.4	22.2 ± 2.0 ^##^
Overweight	27.2 ± 1.5	27.1 ± 1.56	27.9 ± 2.4	27.1 ± 1.1
Obese	32.5 ± 2.2	33.9 ± 4.6	-	32.7 ± 0.0

Categorical variable (gender) is expressed as a percentage, and continuous variables as mean with standard deviation. *n*: number; BMI: body mass index (kg/m^2^). Unpaired *t*-test: ^†††^ *p* <0.001 (Male vs. Female); ^##^ *p* < 0.01 (Italian vs. Spanish female).

**Table 2:** Percentages of drinkers for different alcoholic beverages.

Weekday	Italians		Spaniards	
	Male	Female	Male	Female
	(*n* = 38)	(*n* = 54)	(*n* = 20)	(*n* = 27)
Beer (bottle 33 cl)	%	%	%	%
1, 2 portions	63.2, 5.3	51.9, 3.7	60.0, 5.0	48.2, 18.5
3, 4 portions	-	-	5.0, 5.0	11.1, -
8 portions	-	-		3.7
Total	68.5	55.6	75	81.5
Wine (glass 125 mL)	%	%	%	%
1, 2 portions	47.4, 7.9	57.4, 11.1	40.0, 5.0	40.7, 7.4
3, 4 portions	2.6	-	-	-, 3.7
Total	57.9	68.5	45	51.8
Hard liquor (glass 40 mL)	%	%	%	%
1, 2 portions	21.1, 2.6	24.1, -	30.0, 5.0	22.2, 7.4
3, 4 portions	-	-	5.0, -	7.4, 3.7
Total	23.7	24.1	40	40.7
Cocktail (glass 40 mL)	%	%	%	%
1, 2 portions	21.1, 2.6	35.2, 1.9	20.0, 5.0	14.8, 11.1
8 portions	-	-	-	7.4, 3.7
Total	23.7	37.1	25	37
Saturday night				
Beer (bottle 33 cl)	%	%	%	%
1, 2 portions	57.9, 18.4	35.2, 13.0	25.0, 10.0	14.8, 29.6
3, 4 portions	5.3, -	1.9, -	20.0, 5.0	25.9, 3.7
8 portions	-	-	5	3.7
Total	81.6	50.1	65	77.7
Wine (glass 125 mL)	%	%	%	%
1, 2 portions	29.0, 21.1	44.4, 14.8	30.0, 5.0	22.2, 3.7
3, 4 portions	5.3, 2.6	3.7, 1.9	--	7.4, -
Total	58	64.8	35%	33.30%
Hard liquor (glass 40 mL)	%	%	%	%
1, 2 portions	36.8, 7.9	24.1, -	15.0, 20.0	7.4, 11.1
3, 4 portions	-	-	15.0, 5.0	7.4, 7.4
Total	44.7	24.1	55	33.3
Cocktail (glass 40 mL)	%	%	%	%
1, 2 portions	29.0, 10.5	35.2, 7.4	25.0, 15.0	-, 22.2
3, 4 portions	-	-	5.0, -	11.1, 3.7
8, >8 portions	-	-	-	3.7, 3.7
Total	39.5	42.6	45	44.4

*n*: number; %: percentage. Chi-square test: not significant.

**Table 3:** Alcohol consumption in resident and non-resident students.

Beverage Portions (*n*)	Italians		Spaniards	
Weekday	Resident Students (*n* = 80)	Non-Resident Students (*n* = 12)	Resident Students (*n* = 31)	Non-Resident Students (*n* = 16)
Beer (bottle 33 cl)	1.0 (1.0–1.0)	1.0 (1.0–1.0)	1.0 (1.0–1.5)	1.0 (1.0–2.8)
Wine (glass 125 mL)	1.0 (1.0–1.0)	1.0 (1.0–1.0)	1.0 (1.0–1.0)	1.0 (1.0–1.5)
Hard liquor (glass 40 mL)	1.0 (1.0–1.0) ^##^	-	1.0 (1.0–3.0)	1.0 (1.0–2.0)
Cocktail (glass 40 mL)	1.0 (1.0–1.0) ^##^	-	1.0 (1.0–3.5)	1.0 (1.0–2.0)
Saturday night				
Beer (bottle 33 cl)	1.0 (1.0–2.0) ^###^	1.0 (1.0–1.0)	2.5 (1.3–3.0)	2.0 (1.0–3.0)
Wine (glass 125 mL)	1.0 (1.0–2.0)	1.0 (1.0–2.0)	1.0 (1.0–1.3)	1.0 (1.0–3.0)
Hard liquor (glass 40 mL)	1.0 (1.0–1.0) ^###^	2.0 (2.0–2.0)	2.47 ± 1.06	2.00 ± 0.89
Cocktail (glass 40 mL)	1.0 (1.0–1.0) ^###^	1.5 (1.0–2.0)	2.0 (1.0–3.0)	1.83 ± 0.75

Beverage portions number (*n*) is expressed as median with interquartile range (normality test failed), or as mean with standard deviation (normality test passed). Mann–Whitney test (Italian vs. Spanish resident students): ^##^ *p* <0.01; ^###^ *p* <0.0001.

**Table 4:** Percentage of students who complied with the recommended portion of Mediterranean diet components.

MD Components	Italians		Spaniards	
	Male (*n* = 45) %	Female (*n* = 62) %	Male (*n* = 25) %	Female (*n* = 48) %
Vegetable	26.7	48.4	40.0	47.9
Fruit	22.2	16.1	36.0	27.1
Red meat	40.0	41.9	20.0	31.3
Beans	20.0	22.6	32.0	31.3
Fish	31.1	22.6	32.0	29.2
	Resident students (*n* = 80) %	Non-resident students (*n* = 12) %	Resident students (*n* = 31) %	Non-resident students (*n* = 16) %
Vegetable	39.0 ^##^	33.3	43.8	48.0
Fruit	19.0 ^##^	16.7	33.3	24.0
Red meat	42.1 ^##^	33.3	22.9	32.0
Beans	12.6 ^##^	8.3	39.6	36.0
Fish	21.1 ^##^	25.0	47.9	36.0

MD: Mediterranean diet; *n*: number; %: percentage. Chi-square test: Italian vs. Spanish resident students. ^##^ *p* < 0.01.

**Table 5:** Standardized questionnaires results.

Questionnaires	Italians		Spaniards	
	Male (*n* = 46)	Female (*n* = 62)	Male (*n* = 35)	Female (*n* = 51)
FNS **	22.0 (15.0–27.0)	22.0 (19.0–29.5) ^##^	19.5 (14.3–22.8)	19.0 (14.0–23.5)
MDS-14	6.0 (5.0–7.0)	7.0 (5.0–8.0)	7.0 (4.0–8.0) ^†^	7.0 (6.0–8.0)
MED-55	32.7 ± 4.5 ^‡^	34.5 ± 4.2 ^§^	31.4 ± 5.8 ^§^	33.6 ± 7.3
QueMD	13.0 ± 3.1	13.4 ± 2.7	13.3 ± 2.8	12.9 ± 2.8
aMED *	3.0 (2.0–5.0)	5.0 (3.0–6.0) ^†††^	3.0 (2.0–4.0) ^†^	4.0 (3.0–5.0) ^##^
ntMED	6.0 (4.0–8.0)	6.0 (4.8–8.0)	7.0 (5.8–8.0)	6.0 (5.0–8.0)
AUDIT **	3.0 (2.0–5.0)	3.0 (2.0–4.0) ^##^	3.5 (0.8–8.0)	5.0 (2.5–7.0)

FNS: Food neophobia scale; MDS-14: Mediterranean diet score; MED-55: Mediterranean score; QueMD: Questionnaire to measure Mediterranean diet; aMED: alternate Mediterranean diet score; ntMED: non-typical MD foods score; AUDIT: Alcohol use disorders identification test. Continuous variables are expressed as mean with standard deviation (normal distribution), or as median with interquartile range (normality test failed). Mann–Whitney test: * *p* < 0.05; ** *p* < 0.01 (Italians vs. Spaniards); ^†^ *p* < 0.05; ^†††^ *p* < 0.001 (Male vs. Female); ^##^ *p* < 0.01 (Italian vs. Spanish female). Unpaired *t*-test: ^§^ *p* < 0.05 (Male vs. Female); ^‡^ *p* < 0.05 (Italian vs. Spanish male).

## Data Availability

Data are not available due to ethical restrictions (see Informed Consent Statement).
